# Factors Associated with Migration in Individuals Affected by Leprosy, Maranhão, Brazil: An Exploratory Cross-Sectional Study

**DOI:** 10.1155/2013/495076

**Published:** 2013-09-30

**Authors:** C. Murto, C. Kaplan, L. Ariza, K. Schwarz, C. H. Alencar, L. M. M. da Costa, J. Heukelbach

**Affiliations:** ^1^Swiss Tropical and Public Health Institute, University of Basel, 4002 Basel, Switzerland; ^2^Hamovitch Center for Science in the Human Services, School of Social Work, University of Southern California, 1150 S. Olive Street, Los Angeles, CA 90015, USA; ^3^Department of Community Health, School of Medicine, Federal University of Ceará, 60430-140 Fortaleza, CE, Brazil; ^4^School of Medicine, University of Cologne, Cologne, Germany; ^5^Leprosy Control Program, State Health Secretariat of Maranhão, 65076 Sao Luis, MA, Brazil; ^6^Anton Breinl Centre for Public Health and Tropical Medicine, School of Public Health, Tropical Medicine and Rehabilitation Sciences, James Cook University, Townsville, QLD 4811, Australia

## Abstract

In Brazil, leprosy is endemic and concentrated in high-risk clusters. Internal migration is common in the country and may influence leprosy transmission and hamper control efforts. We performed a cross-sectional study with two separate analyses evaluating factors associated with migration in Brazil's Northeast: one among individuals newly diagnosed with leprosy and the other among a clinically unapparent population with no symptoms of leprosy for comparison. We included 394 individuals newly diagnosed with leprosy and 391 from the clinically unapparent population. Of those with leprosy, 258 (65.5%) were birth migrants, 105 (26.6%) were past five-year migrants, and 43 (10.9%) were circular migrants. In multivariate logistic regression, three independent factors were found to be significantly associated with migration among those with leprosy: (1) alcohol consumption, (2) separation from family/friends, and (3) difficulty reaching the healthcare facility. Separation from family/friends was also associated with migration in the clinically unapparent population. The health sector may consider adapting services to meet the needs of migrating populations. Future research is needed to explore risks associated with leprosy susceptibility from life stressors, such as separation from family and friends, access to healthcare facilities, and alcohol consumption to establish causal relationships.

## 1. Introduction 

Migration has been identified as one of the social determinants influencing transmission dynamics of Neglected Tropical Diseases (NTDs) [1, 2]. In fact, population movement can introduce new diseases when infected migrants move from endemic to nonendemic areas [2, 3]. As strategies of disease control become increasingly important to meet World Health Organization (WHO) standards, a more thorough approach is needed to investigate migration as a risk factor for disease and determine factors associated with migration in a local context.

Migration can influence transmission of NTDs when circumstances influence conditions and risks associated with disease transmission, particularly among the poor who are disproportionately affected [4]. Environmental aspects as a consequence of poverty, such as poor sanitation and overcrowded substandard housing in areas of uncontrolled urbanization [3, 5], as well as lifestyle stressors [6, 7] and behaviors associated with migration [8, 9] can increase susceptibility to infection and disease risk. Many of these factors have also been associated with leprosy transmission [10, 11].

It is estimated that 740 million people are internal migrants, a common condition of life in many low- and middle-income countries [12]. The reasons for migration are numerous and include drivers such as political conflict [13], disaster, and environmental change [14, 15], as well as socioeconomic determinants [13, 16]. While migration is often a strategy to mitigate poverty [17], it is also a means to acquire capital for land, housing, and other opportunities [18, 19]. Movement can be a strategy to realize a higher standard of living, access to better employment, education, and health service infrastructure, primarily between resource poor rural areas and urban centers [5, 13, 17]. These factors can influence short-term temporary or circular migration [17], or permanent relocation [16]. 

In Brazil, migration has historically taken place between poor rural areas in the northeast to large urban centers [20]. Strong social networks between these areas have facilitated ease and cost of movement, important factors in the decision to migrate [12]. A rural exodus of approximately 50 million people occurred between the 1950s and the 1980s, largely from the leprosy endemic Northeastern region. Until the 1970s, rural-urban migration was the result of urban industrialization. Secondarily, rural-rural migration was due to the modernization of agriculture and national policies for frontier expansion in the Amazon region. In the 1980s, severe drought in the Northeast affecting rural agriculture, coupled with severe economic decline throughout the country, influenced outmigration from the region. These decades saw the rapid expansion of urban slums and the expansion of settlements in the Amazon through migration, which has been hypothesized as a possible association with the increased distribution of leprosy in these areas [10, 21]. In urban Rio de Janeiro, a primary destination site for migrants from the Northeast region, leprosy new case detection doubled in the 1980s [22].

The 1990s observed more localized regional migration [23]. While the majority of movement remains between urban centers [20], more recently, there has been a shift toward rural inmigration [20], which can be the result of return or circular migration to these areas. 

As the Brazilian Ministry of Health (MoH) directs its efforts toward leprosy control in areas of high endemic leprosy transmission, interventions targeting high risk and vulnerable groups are an important strategy. Additional protocols should be developed which monitor the effect of population mobility on disease incidence [2, 3] and structure services to meet the needs and behavior of migrants. This development would be important as health systems often are not structured to accommodate migrating populations [2, 17].

The goal of the study reported in this paper was to support the Brazilian MoH, Leprosy Control program in identifying unique factors associated with migration among those with leprosy in an effort to better target services to migrating populations. The study was designed as an exploratory study to investigate factors associated with migration among those newly diagnosed with leprosy in four endemic areas in the Northeast of Brazil, and separately, factors associated with migration among a clinically unapparent population for comparison. More than one-third of the collective population in the research sites in this study were inmigrants, born in areas outside of the municipality and 7% from outside of state of Maranhão [24]. The objectives were to identify demographic, socioeconomic, clinical, and psychosocial factors uniquely associated with migration among those with leprosy, as migration has been identified as a social determinant of health outcomes.

## 2. Materials and Methods

### 2.1. Study Area

**Figure 1:**
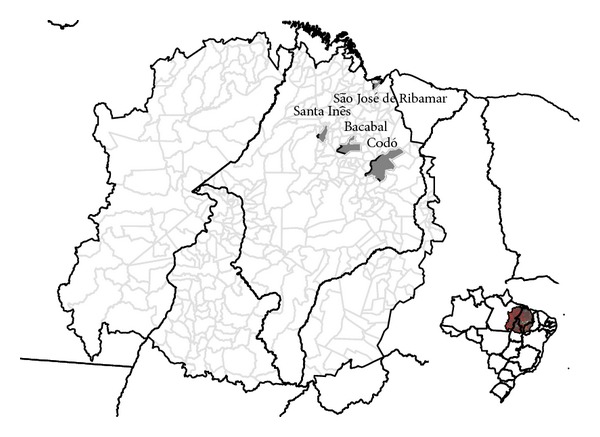
Map of Maranhão and four study sites (Santa Inês, São José de Ribamar, Codó, and Bacabal).

Maranhão state has the third highest annual leprosy case detection rate in Brazil (5.34/10,000 inhabitants in 2010) [25]. The state ranks sixth on the list of outmigration among states and had a circular migration rate of 16.4% between 2004 and 2009 [26]. For this study, four highly endemic municipalities in Maranhão were selected: Santa Inês, São José de Ribamar, Codó, and Bacabal (Figure 1).

Each of these municipalities is located in a major endemic cluster identified by the Brazilian Ministry of Health as high-risk areas for leprosy transmission. Highly endemic clusters were based on national data from 2007 that identified a mean case detection rate of 7.6 per 10,000 residents among 11% of the population [27], well above the WHO elimination goal of <1 per 10,000.

In 2009-2010, Bacabal had an average population size of 99,251 with a leprosy new case incidence of 12.85 per 10,000 inhabitants; Codó, 115,988 inhabitants with new case incidence of 9.40 per 10,000; São José de Ribamar, 151,260 inhabitants and new case incidence of 6.21 per 10,000; and Santa Inês, 81,490 inhabitants and new case incidence of 10.92 per 10,000 [24]. Nearly half of the populations of São José de Ribamar (44.3%) and Santa Inês (45.6%), 29.8% in Bacabal and 17.9% in Codó, were born in other municipalities in Maranhão. Those born outside of the state accounted for 11.2% of the population in Santa Inês, 9% of the population in Bacabal, 8.9% of the population in Codó, and 4.9% in São José de Ribamar [24].

### 2.2. Study Design

**Figure 2:**
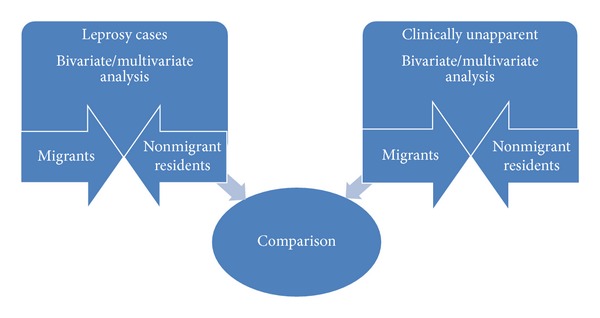
Study design.

This exploratory population-based cross-sectional study was designed to identify factors uniquely associated with migration among those with leprosy compared to nonmigrant residents and included a separate analysis among individuals in a clinically unapparent population without symptoms of leprosy compared to nonmigrant residents. A comparison of factors associated with migration among those with leprosy to the clinically unapparent population was explored. Three dependent measures for migration were defined for both those with leprosy and separately for the clinically unapparent population: (1) migration after birth (municipality of birth different from current municipality of residence), (2) past five-year migration (migrated from a municipality different from the current residence in the last five-years), and (3) past five-year circular migration (past five-year migrants who were currently living in municipality of birth, but migrated to another municipality in the last five-years for a month or more). Migration included all population movement between municipalities, including rural-rural, rural-urban, and urban-urban movement.  Figure 2 highlights the study design which includes bivariate and multivariate analyses conducted separately for birth, past five-year and circular migrants with leprosy compared to nonmigrant residents and similarly for migrants in the clinically unapparent population compared to residents. A detailed analysis of the comparison between migrants with leprosy and clinically unapparent migrants that was collected as part of the larger epidemiological study was beyond the scope of factors associated with migration among those with leprosy and will be explored elsewhere.

### 2.3. Data Collection

Data collection was conducted between April and July 2010 as part of a comprehensive epidemiological study conducted by the MAPATOPI project, an interdisciplinary project to support and improve the Brazilian leprosy control program in a major endemic cluster in the states of Maranhão, Tocantins, Piauí, and Pará, located in the North and Northeast regions of Brazil.

The leprosy population was identified through the database of the National Information System for Notifiable Diseases (*Sistema de Informação de Agravos de Notificação* (SINAN)) available from the Brazilian Ministry of Health, and included new leprosy cases diagnosed in 2009-2010, aged >15 years, and living in the four highly endemic municipalities. A clinically unapparent population without symptoms of leprosy, matched to leprosy cases by age, sex and geographic location, was selected from the *Programa Saúde da Família* (Program for Family Health) and evaluated for leprosy through an extensive clinical exam. Both leprosy cases and those in the clinically unapparent population with a prior history of leprosy or leprosy relapse, living outside of the endemic municipalities and those who could not be located through multiple contact attempts, were excluded.

### 2.4. Field Procedures and Survey Instrument

The Municipality Health Secretariats were informed by the Maranhão State Health Secretariat about the study, and field visits were coordinated for data collection. Patients were invited through community health agents to participate and to be interviewed at the local health care centers. Home visits, often accompanied by local community health agents, were performed when individuals did not present at the health care center or had difficulty attending due to age or disability.

A structured questionnaire was used and composed of seven sections: (1) sociodemographics (sex, age, marital status, education, and employment), (2) housing/economic variables (household density, household income, area of residence, and utility access), (3) clinical/disease-related (clinical form of the disease, operational classification, and grade of disability at diagnosis), (4) health services (visits by community health worker, access to health services), (5) migration (history of migration and length of time at residence), (6) behavior (experienced hunger, alcohol consumption, type, and frequency), and (7) stress (from disease, job/salary loss, divorce/separation, separation from family/friends, and death of close family/friend). Clinical data was also collected from patients' charts. As data from patients' charts was provided through the local healthcare center, in some cases complete data was unavailable. 

### 2.5. Data Analysis

Data were entered twice, using Epi Info software version 3.5.1 (Centers for Disease Control and Prevention, Atlanta, USA), and crosschecked for entry-related errors. Statistical tests were used to assess normality. Data analysis was conducted using STATA version 11 (Stata Corporation, College Station, USA). As a first step, a series of bivariate analyses were conducted examining the significant differences in key theoretical variables for: (i) migrants with leprosy after birth compared to nonmigrant residents with leprosy, (ii) migrants after birth in a clinically unapparent population compared to nonmigrant residents in a clinically unapparent population, (iii) past 5-year migrants with leprosy compared to nonmigrant residents with leprosy, (iv) past 5-year migrants in a clinically unapparent population compared to nonmigrant residents in a clinically unapparent population, (v) past 5-year circular migrants with leprosy compared to nonmigrant residents with leprosy, and (vi) past 5-year circular migrants in a clinically unapparent population compared to nonmigrant residents in a clinically unapparent population. Significant differences (*P* < 0.05) in the hypothesized variables in these analyses were determined with Fisher's exact tests. Odds ratios and 95% confidence intervals were also computed. In the second step, a series of multivariate analyses were executed. The hypothesized variables found to be significant for each migrant group were included in separate multiple logistic regression models for each migrant group controlling for age, sex, and geographic location. A backwards stepwise approach was used to construct these models.

### 2.6. Ethics

The study was approved by the Ethical Review Board of the Federal University of Ceará (Fortaleza, Brazil). Permission to perform the study was also obtained by the Maranhão State Health Secretariat, the State Leprosy Control Program, and municipalities involved. Informed written consent was obtained from study participants after explaining the objectives of the study, and interviews were conducted in private.

## 3. Results

### 3.1. Study Population Characteristics

This study included 394 individuals newly diagnosed with leprosy and 391 individuals from a clinically unapparent population. A total of 135 individuals were not interviewed because they were either not located at their documented place of residence (*n* = 41), had moved (*n* = 34), or had been transferred to a healthcare facility in another municipality after diagnosis (*n* = 18). Another 18 were traveling/away/working, 2 were incarcerated, and 6 were excluded due to mental disability. Others were ill or hospitalized (*n* = 3), or the place of residence was not reachable/outside of municipality (*n* = 4). Only 8 individuals refused to participate.

Of the 394 individuals with leprosy, 215 (54.6%) were males and 179 (45.4%) were females, ranging in age from 15 to 86 years (mean = 42.9 years; standard deviation = 18.8). In the clinically unapparent population (*n* = 391), 216 (55.2%) were males and 175 (44.8%) were females, aged 15 to 89 years (mean = 42.6; standard deviation = 18.8). More than one-third of the individuals with leprosy were working (*n* = 140; 35.5%), and 254 (64.5%) were unemployed, while 159 (40.7%) in the clinically unapparent population were working and 232 (59.3%) unemployed. Nearly half (*n* = 218, 44.7%) of those with leprosy were illiterate, as compared to 137 (35.0%) from the clinically unapparent population. The mean monthly household income was R$924 among those with leprosy and R$906 in the clinically unapparent population. More than one in four among those with leprosy (*n* = 169, 44.4%) and 159 (42.9%) in the clinically unapparent population were living on less than one minimum wage per month (R$551 *≈* USD $296). 

Leprosy-affected individuals included 228 (63.8%) who were classified with multibacillary leprosy. The majority did not have disability at diagnosis (Grade 0) (*n* = 146, 58.2%), which was followed by Grade I (*n* = 82, 32.7%) and Grade II (*n* = 23, 9.2%) disabilities. The clinical form of leprosy was primarily borderline (*n* = 166, 46.9%), followed by tuberculoid (*n* = 82, 23.2%), lepromatous (*n* = 47, 13.3%), indeterminate (*n* = 44, 12.4%), and neural leprosy (*n* = 15, 4.2%). 

Of those with leprosy, 258 (65.5%) were birth migrants, 105 (26.6%) were migrants in the past five-years, and 43 (10.9%) were circular migrants. The clinically unapparent population included 266 (68.0%) birth migrants, 81 (20.7%) past five-year migrants, and 32 (8.2%) circular migrants. 

Variables from the bivariate analysis associated with migration (*P* < 0.05) were included in the multivariate models.

### 3.2. Factors Associated with Migration after Birth

Among birth migrants with leprosy, demographic, behavioral, and clinical factors were found to be associated with migration (Table 1) compared to nonmigrant residents with leprosy (not shown). Behavioral variables included life stressors and alcohol consumption among those with leprosy. Stress as a result of separation from family and friends was associated with migration as was drinking alcohol currently. Not being formally employed by being either employed monthly, daily, or self-employed was significantly associated with migration among those with leprosy as was borderline leprosy diagnosis. Stress from separation from family and friends, never worked, and migration among those 45 and older were found to be associated with migrants in the clinically unapparent population (Table 2) compared to clinically unapparent nonmigrant residents (not shown).

### 3.3. Factors Associated with Migration in the Past Five-Years

Similar to birth migrants, behavioral and lifestyle stressor variables were also associated with migration among those with leprosy (Table 3) compared to nonmigrant residents with leprosy (not shown). Separation from family and friends was significantly associated with migration, as was current alcohol consumption. Two other key factors emerged that differentiated past five-year migrants with leprosy from both birth migrants and migrants in the clinically unapparent population: short length of residence in the current household and difficulty in reaching the healthcare center. Among those in the clinically unapparent population (Table 4), separation from family and friends remained significantly associated with migration when compared to clinically unapparent nonmigrant residents (not shown). Ages 45 and older were no longer associated with past five-year migration in this group. Rather, ages 30 and older were found to be a deterrent to recent migration. Income less than minimum wage (R$511) was also associated with migration in the clinically unapparent population, although no public waste collection, which is sometimes used as a proxy for poverty, was protective.

### 3.4. Factors Associated with Circular Migration Five-Years before Diagnosis

Stressors and behavior were associated with circular migration among those with leprosy (Table 5) compared to nonmigrant residents with leprosy (not shown), consistent with findings among both past five-year and birth migrants. Stress from separation from family and friends was associated with migration among both circular migrants with leprosy compared to nonmigrant residents with leprosy, as well as clinically unapparent migrants (Table 6) compared to clinically unapparent nonmigrant residents (not shown). Unique to migrants with leprosy, current alcohol consumption as well as difficulty in reaching the healthcare center was associated with circular migration. Age 45–59 was a significant deterrent to migration among those with leprosy, while age 60 and older was only marginally protective. 

## 4. Discussion 

The role of social inequalities is in the forefront of Neglected Tropical Diseases (NTDs), underscoring the deep divide that places the most marginalized at highest risk for infection [10, 15]. Among environmental, socioeconomic, and cultural risks, migration is suggested to be a determinant for NTDs [28]. Migration interacts with these factors when fundamental social inequalities determine the necessity to migrate and conditions of migration. This can place migrants at heightened risk for disease while extending disease distribution into new areas. 

In this study we assessed factors associated with migration among those with leprosy in Northeast Brazil. We found several distinct behavioral and psychosocial factors—life stressors, alcohol consumption, and healthcare access—uniquely associated with migration among individuals with leprosy. Alcohol consumption and healthcare access were not associated with migration in a clinically unapparent population, while life stressors were associated with migrant lifestyle regardless of disease.

We examined socioeconomic status, key demographics, and household environment, as these features have been associated with NTDs risk [2, 28] and migration [12, 17]. However, the majority of these social factors were not found to be associated with migration in Maranhão in this exploratory study. This suggests a high level of social homogeneity between nonmigrant residents and migrants residing in the interior and leprosy endemic areas of the state and also may indicate that social features which are prominent in migrating populations are less pronounced when looking at a vulnerable population, such as those affected by leprosy. Household and family exposure to leprosy, the primary exposure risk for leprosy infection, was also not found to be significant when comparing migrants with nonmigrant residents with leprosy. However, future research should consider the role of family and other leprosy contact exposure during migration.

Separation from family and friends, considered a prominent life stressor [29], was found to be significantly associated with migration. Stress can impact psychological well-being and trigger changes to the biological system of the human body and has also been found to be associated with compromised immune response and activation of latent infection for infectious disease [30, 31]. For migrants, stress may render one more vulnerable to infectious diseases such as leprosy and influence symptom onset for those previously exposed.

While stress from separation from family and friends was prominent among migrants regardless of leprosy infection, the odds of this stressor among birth and circular migrants were higher than those of migrants in the clinically unapparent population. Lack of social support that would be more readily available in the home environment can negatively influence the psychological adjustment process among those who migrate [6, 7] and has also been found to increase susceptibility to anxiety and depression among migrants [7]. The collective construction of family in Brazil through extended family and intergenerational participation [32] takes a significant role in Brazilian culture, and in our study, the majority of migrants lived with family members during the past five-year migration period. Poverty, however, has led to increased family separation and interregional migration in Brazil [33], most likely separation from the nuclear family due to cost of migration while maintaining extended familial social networks for employment. Irregular noncontractual employment, as was found among birth migrants, often necessitates separation from family when the cost of migration, particularly to urban areas, cannot accommodate nuclear family movement. Day labour was less likely among those aged 60 and older, and self and monthly employments were more likely among those aged 45–59.

Social stressors can also lead to other behavioral risk such as alcohol abuse [34], and in this study we found that current alcohol consumption differentiates migrants with leprosy from clinically unapparent population. Migration-associated alcohol consumption has been well documented [8, 9, 35] and has also been found to be associated with mycobacterial disease [36] and increased susceptibility to infection [37], which may be relevant in terms of susceptibility to leprosy infection. In Maranhão, current alcohol consumption was found to be a significant factor for those newly diagnosed with leprosy who are birth, past five-year, and circular migrants compared to nonmigrant residents with leprosy. This is particularly concerning among those newly diagnosed with leprosy in terms of interaction with treatment protocols, as alcohol has been found to be a major predictor of leprosy relapse [38], which may stem from the effects of alcohol on the absorption of antibiotics [39]. In addition, liver function among those with more severe lepromatous leprosy is compromised and alcohol consumption should be considered in terms of risk for relapse, susceptibility and disease onset. Younger males in Maranhão were the most likely to drink alcohol which is consistent with other research among migrants [9]. 

Social isolation can accompany those who migrate, particularly in countries such as Brazil where the culture validates the role of family relationships in daily life. Alcohol used as a coping mechanism can be expressed as regular alcohol consumption [35], but also substance abuse [8] and binge drinking [9]. The odds of current alcohol use were fourteen times higher for birth migrants, twice as high for past five-year migrants, and four times higher for circular migrants with leprosy compared to nonmigrant residents with leprosy. Contrarily, alcohol consumption did not differentiate migrants from nonmigrant residents in the clinically unapparent population. 

In Maranhão, past five-year and circular migrants with leprosy had a significantly higher chance of having difficulty reaching the healthcare center than nonmigrant residents with leprosy, while there was no significant difference among migrants and nonmigrant residents in the clinically unapparent population. In fact, healthcare infrastructure is often not compatible to the needs of migrating populations [2, 17]. Long hours of employment and unfamiliarity with new living environments are elements that can affect migrant health access, even when symptoms are evident and persistent. Many municipalities in Brazil have adapted their service availability to some extent, with primary healthcare centers now providing hours that accommodate the working population. 

Distance and illness were the primary reasons for having difficulty in accessing the health clinics among those with leprosy in our study, and other research has also found that migration was a barrier to health facility utilization [7]. Short length of residence among past five-year migrants may partially explain difficulty in reaching the healthcare center, as this could be an additional barrier to access for those who are unfamiliar with the region or lack local social support to locate local services. On a positive note, results are from those currently receiving treatment through their local health center, and another study found that migration is not a barrier to treatment interruption in Brazil after diagnosis [40]. It is nonetheless relevant to point out that illness and distance are significantly higher for migrants than for nonmigrant residents with leprosy, and failing to reach migrating populations may hinder control efforts [41].

The three main factors—lifestyle stress, alcohol consumption, and healthcare access—present important considerations in the role of risks associated with migration and consequently disease control. In their advanced expression, alcohol abuse and significant psychological distress not only affect immune system response but can also contribute to delay in accessing health services, late diagnosis, and treatment interruption [7, 42]. Thus, it continues to be important to identify mechanisms and adaptations for leprosy control efforts to respond to unique risks associated with migration.

There are some indications that advanced age of 45 or older is a deterrent to migration among both those with leprosy and the clinically unapparent population. A positive association with migration and advanced age among birth migrants in the clinically unapparent population is likely reflective of historic population movement in the 1980s in Brazil, as this is no longer significant in recent past five-year and circular migration. 

## 5. Limitations

We believe that our study highlights and is representative of national issues surrounding leprosy and migration in Brazil as (1) the population sample stemmed from endemic municipalities inside of clusters at high-risk for transmission and (2) the state of Maranhão is among the top states in Brazil with significant in- and outmigration. However, the study presented is a cross-sectional study limited to four municipalities in a hyperendemic area and thus subject to limitations. For example, outmigrants from the study sites were not included in this study and only data for migrants which are currently present in these sites compared to nonmigrant residents was included. Socioeconomic data only concerned the point in time when the research was conducted and thus excludes the timeframe when migration occurred. Data may not reflect the time of actual leprosy infection given a five-year average latency period. Data on stress factors pertained to the past five-years and could include stress during the postinfection time period. 

Due to difficulties in establishing the sequence of events, interpretation of causal relationship should be taken with care. Events may be caused or compounded by migration and/or cause migration itself. Important indirect factors relevant to leprosy transmission need to be also considered in future research on migration and leprosy in highly endemic municipalities.

## 6. Conclusions 

This is the first systematic cross-sectional study focusing on migration among people affected by leprosy. Psychosocial factors and healthcare access emerged as factors significantly associated with migration in this vulnerable population, in contrast to a clinically unapparent population. Findings point to the opportunity to assist migrants in maintaining ties to their home communities, thus not only potentially reducing stressors with separation from family and friends, but also potentially influencing the role of this separation as it may affect alcohol consumption. We included a discussion of both alcohol consumption and stressors as they pertain to reduced immune function and psychological well-being cited in the literature to support our findings; however, limited research still exists on the role of alcohol consumption and NTDs susceptibility. Further qualitative and quantitative longitudinal research addressing mild, moderate, and severe alcohol consumption could establish causal relationships and also explore the role of stress and substance use as risks for leprosy infection. Additionally, the role of acute stress through long-term migration and repetitive stress through circular migration could be explored.

As healthcare access emerged as a primary concern for migrants with leprosy, the health sector may consider restructuring services to meet the needs of migrating populations. Extended hours of operation, such as evening and weekend hours would be an important first step in this regard. Additionally, assistance with transportation to clinical facilities or providing mobile medical care could potentially alleviate problems associated with access. Facilitating the ease of healthcare access may increase early diagnosis and thus reduce advanced disability and improve medication adherence, while also supporting leprosy elimination and control in Brazil. 

## Figures and Tables

**Table 1:** Multivariate analysis of factors associated with migration after birth among migrants diagnosed with leprosy compared to nonmigrant residents with leprosy.

Migration after birth			
Leprosy cases			
	Migrants^†^ *N* = 258 (66.2%)	AOR (95% CI)*	*P*
Worker contract status (employed)			
Formally employed	18 (45.0%)	1.0	
Self-employed	13 (68.4%)	15.27 (1.44–161.69)	**0.02**
Monthly employment	14 (73.7%)	8.83 (1.53–50.81)	**0.02**
Day labour	43 (74.1%)	10.35 (2.59–41.31)	**0.001**
Alcohol consumption			
Never drank	54 (53.5%)	1.0	
Drink currently	38 (67.9%)	14.53 (1.64–128.31)	**0.02**
Drank in past 5 yrs	120 (68.6%)	5.65 (0.95–33.45)	0.56
Stopped drinking >5 years ago	41 (80.4%)	6.69 (0.65–69.15)	0.11
Difficulty to reach the healthcare center			
Yes	73 (76.8%)	0.91 (0.20–4.17)	0.91
No	184 (62.8%)	1.0	
Stress separated from family/friends			
Yes	57 (78.1%)	7.64 (1.25–46.71)	**0.03**
No	200 (63.3%)	1.0	
Stress job/salary loss			
Yes	77 (77.0%)	0.92 (0.25–3.48)	0.91
No	180 (62.3%)	1.0	
Leprosy diagnosis			
Tuberculoid	48 (59.3%)	4.36 (0.79–24.11)	0.09
Borderline	123 (75.5%)	5.41 (1.01–29.14)	**0.049**
Lepromatous	27 (57.5%)	0.15 (0.02–1.33)	0.09
Indeterminate	22 (50.0%)	1.0	
Neural	9 (60.0%)	0.84 (0.03–21.87)	0.92

^†^Data not available for all individuals, significant results at 95% (*P* < 0.05) are highlighted in bold, *adjusted odds rates (AOR) are only presented for those variables included in the final regression model.

**Table 2:** Multivariate analysis of factors associated with migration after birth among migrants in a clinically unapparent population without symptoms of leprosy compared to clinically unapparent non-migrant residents.

Migration after birth			
Clinically unapparent population			
	Migrants^†^ *N* = 266 (68.4%)	AOR (95% CI)*	*P*
Age-groups (yrs)			
15–29	71 (55.0%)	1.0	
30–44	54 (64.3%)	1.20 (0.65–2.2)	0.56
45–59	67 (77.9%)	2.4 (1.15–5.01)	**0.02**
≥60	74 (82.2%)	3.08 (1.3–7.31)	**0.01**
Sex			
Male	157 (72.7%)	1.27 (0.79–2.03)	0.32
Female	109 (63.0%)	1.0	
Education			
No formal education	107 (78.7%)	1.20 (0.62–2.34)	0.59
Some education	159 (62.9%)	1.0	
Life occupation			
Farmer	105 (76.6%)	0.80 (0.43–1.49)	0.48
Never worked	20 (40.0%)	0.39 (0.2–0.78)	**0.01**
Other work	139 (69.5%)	1.0	
Stress separated from family/friends	55 (78.6%)	2.35 (1.22–4.51)	
Yes	55 (78.6%)	2.35 (1.22–4.51)	**0.01**
No	211 (66.1%)	1.0	

^†^Data not available for all individuals, significant results at 95% (*P* < 0.05) are highlighted in bold, *adjusted odds rates (AOR) are only presented for those variables included in the final regression model.

**Table 3:** Multivariate analysis of factors associated with past 5 year migration among migrants diagnosed with leprosy compared to non-migrant residents with leprosy.

Past 5-year migration			
Leprosy cases			
	Migrants^†^ *N* = 105 (26.7)	AOR (95% CI)*	*P*
Age-groups (yrs)			
15–29	43 (33.6%)	1.0	
30–44	29 (33.7%)	0.75 (0.35–1.58)	0.45
45–59	19 (21.8%)	0.57 (0.24–1.3)	0.28
≥60	14 (15.2%)	0.45 (0.16–1.31)	0.14
Education			
No formal education	34 (19.4%)	0.66 (0.29–1.49)	0.32
Some education	71 (32.6%)	1.0	
Head of household education			
No formal education	42 (20.4%)	0.90 (0.43–1.85)	0.77
Some education	54 (32.5%)	1.0	
Life occupation			
Farmer	31 (21.2%)	0.83 (0.41–1.68)	0.60
Never worked	12 (23.5%)	0.77 (0.29–2.02)	0.59
Other work	62 (31.0%)	1.0	
Electricity			
No	4 (80.0%)	14.75 (1.09–199.83)	0.43
Yes	101 (26.0%)	1.0	
Length of time in current residence			
0–4 years	61 (43.0%)	2.51 (1.37–4.63)	**0.003**
5–10 years	12 (17.4%)	0.74 (0.31–1.77)	0.50
≥11 years	30 (17.7%)	1.0	
Alcohol consumption			
Never drank	18 (17.7%)	1.0	
Drink currently	19 (33.9%)	2.52 (1.01–6.28)	**0.047**
Drank in past 5 yrs	56 (31.8%)	1.88 (0.87–4.07)	0.12
Stopped drinking >5 yrs ago	10 (19.6%)	1.40 (0.48–4.11)	0.54
Difficulty to reach the healthcare centre			
Yes	36 (37.9%)	2.23 (1.22–4.09)	**0.01**
No	69 (23.3%)	1.0	
Stress job/salary loss			
Yes	38 (37.3%)	1.54 (0.81–2.94)	0.19
No	67 (23.1%)	1.0	
Stress divorce/separated			
Yes	26 (37.7%)	0.76 (0.35–1.63)	0.48
No	79 (24.5%)	1.0	
Stress separated from family/friends			
Yes	34 (46.0%)	2.64 (1.36–5.10)	**0.004**
No	71 (22.3%)	1.0	

^†^Data not available for all individuals, significant results at 95% (*P* < 0.05) are highlighted in bold, *adjusted odds rates (AOR) are only presented for those variables included in the final regression model.

**Table 4:** Multivariate analysis of factors associated with past 5 year migration among migrants in a clinically unapparent population without symptoms of leprosy compared to clinically unapparent non-migrant residents.

Past 5-year migration			
Clinically unapparent population			
	Migrants^†^ *n* = 81 (20.7)	AOR (95% CI)*	*P*
Age-groups (yrs)			
15–29	41 (31.3%)	1.0	
30–44	22 (26.2%)	0.72 (0.34–1.55)	**0.02**
45–59	11 (12.8%)	0.3 (0.11–0.84)	**0.02**
≥60	7 (7.8%)	0.23 (0.07–0.78)	**0.01**
Education			
No formal education	15 (11.0%)	1.04 (0.35–3.1)	0.9
Some education	66 (26.0%)	1.0	
Head of household education			
No formal education	22 (12.5%)	0.61 (0.27–1.4)	0.25
Some education	53 (26.2%)	1.0	
Life occupation			
Farmer	18 (13.1%)	1.57 (0.66–3.72)	0.3
Never worked	13 (26.0%)	0.94 (0.38–2.32)	0.89
Other work	49 (24.3%)	1.0	
Home ownership			
No	10 (55.6%)	3.34 (0.99–11.25)	0.05
Yes	71 (19.1%)	1.0	
Household monthly income^††^			
>511 R$	54 (25.5%)	1.0	
0–511 R$	26 (16.4%)	2.15 (1.09–4.23)	**0.02**
Public waste collection			
No	11 (11.9%)	0.43 (0.19–0.97)	**0.03**
Yes	70 (23.7%)	1.0	
Stress separated from family/friends			
Yes	34 (48.6%)	5.76 (2.96–11.22)	<**0.0001**
No	47 (14.6%)	1.0	

^†^Data not available for all individuals, ^††^at the time of the survey 1 US$ was equivalent to 1.72 R$, and R$511, the official minimum wage as set by the Federal Government, significant results at 95% (*P* < 0.05) are highlighted in bold, *adjusted odds rates (AOR) are only presented for those variables included in the final regression model.

**Table 5:** Multivariate analysis of factors associated with past 5 year circular migration among migrants diagnosed with leprosy compared to non-migrant residents with leprosy.

Past 5-year circular migration			
Leprosy cases			
	Migrants^†^ *n* = 43 (24.57)	AOR (95% CI)*	*P*
Age-groups (years)			
15–29	25 (32.1%)	1.0	
30–44	13 (31.7%)	0.82 (0.29–2.29)	0.7
45–59	3 (10.0%)	0.17 (0.04–0.79)	**0.02**
≥60	2 (7.7%)	0.1 (0.01–1.12)	0.06
Education			
No formal education	10 (15.9%)	0.84 (0.25–2.8)	0.77
Some education	33 (29.5%)	1.0	
Head of household education			
No formal education	15 (17.1%)	0.57 (0.22–1.49)	0.26
Some education	25 (32.5%)	1.0	
Alcohol consumption			
Never drank	6 (11.3%)	1.0	
Drink currently	12 (40.0%)	4.46 (1.3–15.34)	**0.02**
Drank in past 5 years	22 (28.6%)	2.26 (0.7–7.29)	0.17
Stopped drinking >5 years ago	2 (16.7%)	2.47 (0.27–22.92)	0.43
Difficulty to reach the healthcare centre			
Yes	16 (42.1%)	2.72 (1.07–6.93)	**0.04**
No	27 (19.9%)	1.0	
Time to Diagnosis			
<7 days	25 (25.3%)	1.0	
7–30 days	11 (29.0%)	1.14 (0.41–3.17)	0.8
30–60 days	1 (20.0%)	1.39 (0.1–19.1)	0.81
>60 days	6 (20.7%)	1.18 (0.33–4.2)	0.8
Stress separated from family/friends			
Yes	14 (46.7%)	4.71 (1.66–13.41)	**0.004**
No	29 (20.0%)	1.0	

^†^Data not available for all individuals, significant results at 95% (*P* < 0.05) are highlighted in bold, *adjusted odds rates (AOR) are only presented for those variables included in the final regression model.

**Table 6:** Multivariate analysis of factors associated with past 5 year circular migration among migrants in a clinically unapparent population without symptoms of leprosy compared to clinically unapparent non-migrant residents.

Past 5 year circular migration			
Clinically unapparent population			
	Migrants^†^ *n* = 32 (20.65)	AOR (95% CI)*	*P*
Life occupation			
Farmer	7 (18.0%)	0.55 (0.21–1.5)	0.23
Never worked	3 (9.1%)	0.27 (0.08–1.03)	0.06
Other work	22 (26.5%)	1.0	
Stress separated from family/friends			
Yes	10 (40.0%)	3.36 (1.3–8.66)	**0.01**
No	22 (16.9%)	1.0	

^†^Data not available for all individuals, significant results at 95% (*P* < 0.05) are highlighted in bold, *adjusted odds rates (AOR) are only presented for those variables included in the final regression model.
